# Effects of 39 Compounds on Calmodulin-Regulated Adenylyl Cyclases AC1 and *Bacillus anthracis* Edema Factor

**DOI:** 10.1371/journal.pone.0124017

**Published:** 2015-05-06

**Authors:** Carolin Lübker, Roland Seifert

**Affiliations:** Institute of Pharmacology, Hannover Medical School, Hannover, Germany; The Ohio State University, UNITED STATES

## Abstract

Adenylyl cyclases (ACs) catalyze the conversion of ATP into the second messenger cAMP. Membranous AC1 (AC1) is involved in processes of memory and learning and in muscle pain. The AC toxin edema factor (EF) of *Bacillus anthracis* is involved in the development of anthrax. Both ACs are stimulated by the eukaryotic Ca^2+^-sensor calmodulin (CaM). The CaM-AC interaction could constitute a potential target to enhance or impair the AC activity of AC1 and EF to intervene in above (patho)physiological mechanisms. Thus, we analyzed the impact of 39 compounds including typical CaM-inhibitors, an anticonvulsant, an anticholinergic, antidepressants, antipsychotics and Ca^2+^-antagonists on CaM-stimulated catalytic activity of AC1 and EF. Compounds were tested at 10 μM, i.e., a concentration that can be reached therapeutically for certain antidepressants and antipsychotics. Calmidazolium chloride decreased CaM-stimulated AC1 activity moderately by about 30%. In contrast, CaM-stimulated EF activity was abrogated by calmidazolium chloride and additionally decreased by chlorpromazine, felodipine, penfluridol and trifluoperazine by about 20–40%. The activity of both ACs was decreased by calmidazolium chloride in the presence and absence of CaM. Thus, CaM-stimulated AC1 activity is more insensitive to inhibition by small molecules than CaM-stimulated EF activity. Inhibition of AC1 and EF by calmidazolium chloride is largely mediated via a CaM-independent allosteric mechanism.

## Introduction

Adenylyl cyclases (AC) catalyze the conversion of ATP into the second messenger cAMP, which is involved in the regulation of numerous processes such as hormone secretion and cardiac contractility [1, 2]. AC isoform 1 (AC1) is one of nine isoforms of membranous ACs [3]. AC1 is expressed in brain and is involved in physiological processes of memory and learning [4–9]. *Bacillus anthracis* is the causative agent of anthrax, a potentially lethal infectious disease. The AC toxin edema factor, released by *Bacillus anthracis* during infection, is involved in the pathogenesis of anthrax and facilitates bacterial growth via inhibiting the innate immune system by generating extremely high cAMP levels [10–12].

Both AC1 and EF are stimulated by the eukaryotic Ca^2+^-sensor calmodulin (CaM) [13–15]. The three dimensional structure of CaM is modified by binding four Ca^2+^-ions. Ca^2+^-saturated CaM possesses a flexible linker region, connecting a C-terminal and an N-terminal globular region [16], which affords the interaction with numerous target proteins like myosin light-chain kinase (MLCK), cyclic nucleotide phosphodiesterase (PDE) and *Bordetella pertussis* AC toxin CyaA (CyaA) and the associated regulation of diverse physiological processes [16–21].

Numerous small molecules were identified as inhibitors of CaM-target interactions [22–26]. Especially antipsychotics but also antidepressants, antihistaminics, antimalarials, smooth muscle relaxants and anticholinergics inhibit CaM-target interaction via binding to CaM [25–39]. Most antipsychotics are antagonists at biogenic amine G-protein coupled receptors, and most antidepressants inhibit neuronal biogenic amine uptake, but the specific mechanisms by which the clinical effects are mediated are not known. CaM is a fundamental biochemical regulator via interacting with a wide variety of proteins [16, 26] why inhibition of CaM could be a common mechanism for establishing some of the pharmacological effects of these drugs [25, 26, 33, 39, 40]. Thus, CaM possibly consitutes an important target for pharmacological intervention [26, 39, 41].

Most compounds identified as potent CaM-inhibitors share common structural features: an amphiphatic amine coupled to large hydrophobic regions. Thereby, two aromatic rings are ideal whereas only one aromatic ring in the structure is adverse [26]. Substituents increasing the lipid solubility are beneficial as well as the location of the amino group at intervals of three-carbon atoms removed from the ring [26]. A positive charge is necessary to interact with the negatively charged CaM [26]. Altogether, a hydrophobic interaction combined with an electrostatic interaction between CaM and the small molecule in a particular orientation is required for a potent CaM-inhibition [26].

The phenothiazine antipsychotics such as chlorpromazine, fluphenazine, promethazine, thioridazine and trifluoperazine possess above structural features and are potent CaM-inhibitors. The mechanism of their binding to CaM is well studied. The drugs bind directly to CaM in a Ca^2+^-dependent manner whereby one to three binding sites per CaM are existant [35–38, 40, 42–44]. Via CaM-binding, phenothiazine antipsychotics inhibit diverse enzymes (e.g. ACs, plasma membrane Ca^2+^-ATPase (PMCA), MLCK, PDE and phospholipase A_2_) and processes (e.g. α-adrenergic response, Ca^2+^-uptake, catecholaminergic function, insulin release, leukocyte function, neurotransmitter release, smooth muscle contraction) (summarized in [26]). Diphenylbutylpiperidine antipsychotics and thioxanthene anticholinergics are also potent CaM-inhibitors in accord to the structural features required for potent CaM-inhibition [26]. In contrast, butyrophenone antipsychotics such as haloperidol, containing only one aromatic ring, are not in accordance with above structural features and they are not as potent as phenothiazines, diphenylbutylpiperidines or thioxanthenes in terms of CaM-inhibition [26]. Butyrophenone antipsychotics are highly potent with regard to the antipsychotic activity. Antipsychotic activity and CaM-inhibition are not necessarily related as expected based on findings for phenothiazine, diphenylbutylpiperidine and thioxanthene antipsychotics [25, 32, 40, 45]. Tricyclic antidepressants are also potent CaM-inhibitors as demonstrated by studies analyzing CaM activation of PDE [25]. At first glance, the potent CaM-inhibitor *N*-(6-aminohexyl)-5-chloro-1-naphthalenesulfonamide (W-7) appears to differ from the proposed structure but, in fact, the naphthalene ring constitutes the large hydrophobic region and a side-chain amino group in proposed distance removed from the ring is also present [29, 31].

Ca^2+^-antagonists such as diltiazem, felodipine and verapamil were studied as an additional class of drugs with regard to CaM-inhibition. Fluorescence studies using dansylated CaM identified diltiazem and verapamil as low-potency CaM-antagonists, whereas felodipine binds to CaM with high affinity, similar to trifluoperazine and W-7 [46, 47]. Finally, calmidazolium chloride (CDZ) is known as a potent CaM-inhibitor [48], investigated in numerous studies with varying CaM-targets. The structure of CDZ substantially differs from structures of antipsychotics. In fact, CDZ is more bulky and complex with its four aromatic rings including an imidazolium. For some CaM-targets (e.g. CyaA and sarcoplasmatic/endoplasmatic reticulum Ca^2+^-ATPase (SERCA)), where CDZ acts as a potent inhibitor, it was suggested that the potency of inhibition is correlated with the hydrophobicity and the size of the inhibitor [21, 49]. In contrast to most of above CaM-inhibitors, CDZ inhibits many CaM-targets in a CaM-independent manner [18, 21, 49–52]. Table 1 summarizes the effects of CaM-inhibitors on CaM-target interactions. Concentrations of CaM-inhibitors used in these studies were extremely high (up to 1000 μM) and far beyond therapeutic plasma concentrations of antipsychotics and antidepressants (up to 10 μM) [53]. In order to investigate the impact of small molecules on CaM-AC1 and CaM-EF interaction, a small library out of above described CaM-inhibitors and related compounds at a concentration of 10 μM was analyzed in this study. Small molecules used are classified in Table 2.

**Table 1:** Effects of important CaM-inhibitors on particular CaM-target interactions.

CaM-target and reference	Assays used	Analyzed substances, used concentraction ranges^a^ and IC_50_ values^b^	Most important findings
AC in rat cerebellar membranes [70]	AC activity assay	CDZ (1–80 μM) *IC*_*50*_: *3 μM*; TFP (10–100 μM) *IC*_*50*_: *30 μM*; W-7 (10–100 μM)	CDZ and TFP inhibit AC in an apparently competitive manner, W-7 in a non-competitive. Potency of antagonists is dependent upon CaM-concentration. CaM-AC complex is relatively refractory to inhibition.
AC2, AC9 and recombinant AC fusion proteins [51]	AC activity assay	CDZ (1–1000 μM) *IC*_*50*_ *(AC5-AC2 fusion protein)*: *20 μM*	CDZ is a non-competitive inhibitor of AC activity. Effect of CDZ is mediated by direct interaction with the catalytic core of AC in an apparently different manner than inhibition by adenosine analogues. CDZ has biphasic effects on AC activity: at low concentrations of CDZ, AC activity increases.
*Bordetella pertussis* AC toxin CyaA [21]	AC activity assay, fluorescence studies	CDZ (0.001–100 μM) *IC*_*50*_: *0*.*66 μM*; TFP (0.001–100 μM); W-7 (0.001–100 μM)	Inhibition of CyaA by CDZ is CaM-independent. Data suggest that CDZ binds to one or two hydrophobic binding sites in CyaA preventing conformational changes required for catalytic activity of CyaA. TFP and W-7 do not inhibit CyaA.
NO-activated sGC (cerebellar cells and purified enzyme) [52]	Ca^2+^-imaging, NO and sGC activity assay	CDZ (1–100 μM) *IC*_*50*_: *11*.*3 μM*; TFP (3–300 μM) *IC*_*50*_: *177 μM*	Inhibitory effect of CDZ on cGMP accumulation does not depend on Ca^2+^-signaling. CDZ directly inhibits purified sGC in an uncompetitive and CaM-independent manner. CDZ inhibits purified sGC with similar potency to its effect on cerebellar astrocytes.
SERCA [49]	Ca^2+^-ATPase activity assay, fluorescence studies, Ca^2+^-binding and phosphoryla-tion studies	Various concentrations up to 200 μM were used of CaM-binding peptide *IC*_*50*_: *7*.*0 μM*; CDZ *IC*_*50*_: *0*.*5 μM*; chlorpromazine *IC*_*50*_: *23 μM*; fluphenazine *IC*_*50*_: *15 μM*; TFP *IC*_*50*_: *45 μM*; W-7 *IC*_*50*_: *125 μM*	Effects of CaM-antagonists are independent of CaM and they inhibit the SERCA in an isoform-specific manner. CaM-antagonists not only reduce the maximal activity, they also increase the K_m_ for Ca^2+^-binding of the high-affinity (stimulatory) side. CDZ and CaM-binding peptide are the most potent inhibitors of SR^c^ Ca^2+^-ATPase activity, W-7 is the least potent inhibitor.
Skeletal muscle SR^c^ Ca^2+^-ATPase [50]	Ca^2+^-ATPase activity assay, steady-state phosphoryla-tion assay	CDZ (1–10 μM) *IC*_*50*_: *4*.*1 μM*; TFP (10–100 μM) *IC*_*50*_: *~ 200 μM*	CDZ inhibits skeletal SR^c^ Ca^2+^-ATPase with high affinity and in a non-competitive manner. Results suggest that inhibition is not a specific antagonism of enzyme activation by CaM but depends on binding to the membrane phospholipids.
a) Ca^2+^/CaM-dependent PDE; b) trypsin-treated PDE: lost its sensitivity to Ca^2+^/CaM [17]	Radiometric PDE activity assay, binding study with W-7-coupled sepharose	Chlorpromazine *a) IC*_*50*_: *35 μM*, *IC*_*50*_: *210 μM*; TFP (10–1000 μM) *a) IC*_*50*_: *7 μM*, *IC*_*50*_: *110 μM*; W-7 (10–1000 μM) *a) IC*_*50*_: *28 μM*, *b) IC*_*50*_: *375 μM*	CaM-antagonists inhibit also trypsin-treated PDE. Binding sites for CaM-antagonists on trypsin-treated PDE have structural similarities to Ca^2+^/CaM. CaM-antagonists binding site is at or near the active site.
Connexin50 gap junctions (expressed in the lens of the eye) [78]	Whole cell patch clamp experiments, NMR studies, fluorescence studies, CD, mass spectrometry	Various concentrations of CDZ and Cx50-peptide (Cx50p^141-166^ out of the CaM-binding domain of Cx50)^d^	Shown by using CDZ and Cx50p^141-166^: Ca^2+^-dependent inhibition of Cx50 gap junctions is mediated by CaM.
MLCK [18]	MLCK activity assay, fluorescence studies	CDZ (0.3–500 μM) *IC*_*50*_: *18 μM* ^e^; TFP (0.3–500 μM) *IC*_*50*_: *144 μM* ^e^; W-7 (0.3–500 μM) *IC*_*50*_: *300 μM* ^e^	CDZ, TFP and W-7 inhibit the MLCK CaM-dependently and CaM-independently. All antagonists bind to MLCK. CaM-independent inhibition of antagonists occurred by binding to MLCK.

^a^Concentration ranges studied are given in parentheses.

^b^If available, IC_50_ values are indicated in italics.

^c^SR, sarcoplasmatic reticulum.

^d^IC_50_ values were not determined.

^e^IC_50_ values obtained from experiments using 115 nM CaM and 8.0 mg/ml MLCK.

**Table 2:** Classification of analyzed small molecules.

Classification	Compound	Structural class
**Anticholinergic**	Metixene	Thioxanthene
**Anticonvulsant**	Carbamazepine	Dibenzazepine
**Antidepressants**	Amitriptyline	Tricyclic; Dibenzocycloheptadiene Dibenzocycloheptadien
	Amoxapine	Tricyclic; Dibenzoxazepine
	Clomipramine	Tricyclic; Dibenzazepine
	Desipramine	Tricyclic; Dibenzazepine
	Dibenzepin	Tricyclic; Dibenzdiazepine
	Lofepramine	Tricyclic; Dibenzazepine
	Maprotiline	Tetracyclic; Anthracene
	Mianserin	Tetracyclic; Dibenzpyrazinoazepine
	Nortriptyline	Tricyclic; Dibenzcycloheptene
	Opipramol	Tricyclic; Dibenzazepine
	Paroxetine	Selective serotonin reuptake inhibitor; Piperidine
	Protriptyline	Tricyclic; Dibenzannulene
	Trimipramine	Tricyclic; Dibenzazepine
**Antipsychotics**	Chlorprothixene	Typical; Thioxanthene
	Chlorpromazine	Typical; Phenothiazine
	Clozapine	Atypical; Dibenzodiazepine
	Clozapine *N*-oxide	Active metabolite of clozapine
	*N*-Desmethylclozapine	Active metabolite of clozapine
	Fluphenazine	Typical; Phenothiazine
	Haloperidol	Typical; Butyrophenone
	Levomepromazine	Typical; Phenothiazine
	Loxapine	Typical; Dibenzoxazepine
	Mesoridazine	Typical; Phenothiazine
	Olanzapine	Atypical; Benzodiazepine
	Penfluridol	Typical; Diphenylbutylpiperidine
	Perphenazine	Typical; Phenothiazine
	Prochlorperazine	Typical; Phenothiazine
	Promethazine	Typical; Phenothiazine
	Risperidone	Atypical; Pyrimidine
	Sulforidazine	Active metabolite of thioridazine
	Thioridazine	Typical; Phenothiazine
	Trifluoperazine	Typical; Phenothiazine
**Ca** ^**2+**^ **-antagonists**	Diltiazem	Benzothiazepine
	Felodipine	1,4-Dihydropyridine
	Verapamil	Phenylalkylamine
**Other CaM-inhibitors**	Calmidazolium chloride	Imidazolium
	W-7	Naphthalenesulfonamide

## Materials and Methods

### Materials

*Spodoptera frugiperda* (Sf9) insect cells for expression of ACs were from American Type Culture Collection (Rockville, MD, USA). Insect-XPRESS Media and gentamicin sulfate for Sf9 cell culture were acquired from Lonza (Basel, Switzerland). 1x Dulbecco’s phosphate buffered saline without Ca^2+^ and Mg^2+^ was purchased from PAA laboratories (Pasching, Austria). Baculovirus encoding bovine AC1 was kindly provided by Prof. Alfred G. Gilman (University of Texas, Dallas, TX, USA) [15]. [α-^32^P]ATP (3,000 Ci/mmol) was purchased from Hartmann (Braunschweig, Germany). Aluminium oxide, MP Alumina N Super I, was obtained from MP Biomedicals (Eschwege, Germany). All other materials used for AC activity assay were from sources as previously described [54]. Bovine serum albumin was purchased from Sigma-Aldrich (St. Louis, MO, USA). Bio-Rad DC protein assay kit was purchased from Bio-Rad (Hercules, CA, USA). Sources of materials for EF expression and purification were as previously decribed [55]. The small molecules were obtained from Sigma-Aldrich (Taufkirchen, Germany), Biotrend (Köln, Germany), RBI (Natick, MA, USA), Tocris (Bristol, United Kingdom) and Calbiochem (Darmstadt, Germany). CaM-wt was kindly provided by Prof. Jeffrey L. Urbauer and Ramona J. Bieber Urbauer, University of Georgia, GA, USA and purified EF by Prof. Wei-Jen Tang, University of Chicago, IL, USA.

### Expression of membranous ACs in Sf9 cells and preparation of cell membranes

Infection of Sf9 insect cells with baculovirus harbouring the gene encoding membranous AC1 and preparation of ACs in cell membranes were essentially performed as previously described [56]. Sf9 cells were cultured in Insect-XPRESS media supplemented with 100 μg/ml gentamicin and 5% (v/v) fetal bovine serum at 28°C. For expression of membranous ACs, 3 x 10^6^ cells/ml were infected with a dilution of 1:100 of high-titer baculovirus. The cell suspension was incubated for 48 h at 28°C. For preparation of membranes containing ACs, harvested cells were washed with 1x Dulbecco’s phosphate buffered saline without Ca^2+^ and Mg^2+^. After centrifugation at 1,000 × g for 10 min at 4°C, the cell pellet was resuspended in lysis buffer, pH 7.4 (10 mM Tris-HCl, 1 mM EDTA and 10 μg/ml benzamide, 10 μg/ml leupeptin and 200 μM phenylmethanesulfonyl fluoride as protease inhibitors). Cells were lysed with 25 strokes in a dounce homogenizer. A centrifugation step at 500 × g for 5 min at 4°C was conducted to sediment the nuclei. The supernatant suspension containing the cell membranes was centrifuged at 40,000 × g for 20 min at 4°C. The supernatant fluid was discarded. At this point CaM was removed from the membranes by Ca^2+^-chelation using EGTA. The cell pellet was resuspended and incubated for 10 min at 4°C in HEED buffer, pH 7.4 [15] (20 mM HEPES, 1 mM EDTA, 1 mM EGTA, 2 mM DTT and 10 μg/ml benzamide, 10 μg/ml leupeptin and 200 μM phenylmethanesulfonyl fluoride as protease inhibitors) as previously described [15,57,58]. Following incubation, membranes were centrifuged at 40,000 × g for 20 min at 4°C. The supernatant fluid was discarded and the cell pellet was resuspended again in lysis buffer. A final centrifugation step was performed at 40,000 × g for 20 min at 4°C. The supernatant fluid was discarded and the cell pellet was resuspended in binding buffer, pH 7.4 (75 mM Tris-HCl, 12.5 mM MgCl_2_ and 1 mM EDTA). The protein concentration of membrane preparations was determined by using the Lowry method [59] with the Bio-Rad DC protein assay kit using bovine serum albumin as the standard. Membranes were stored at -80°C.

### Expression and purification of EF

The plasmid pProExH6-EF was prepared and amplified in *E*. *coli* BL21 (DE3)/pUBS520 cells as previously described [60–63]. Expression and purification of EF via DEAE, SP-Sepharose and Ni-NTA-columns were performed as previously described [61] with minor modifications [55].

### Expression and purification of CaM

CaM was expressed in *E*. *coli* and purified as previously described [64]. The CaM gene subcloned into the *E*. *coli* expression vector is the chicken gene (Uni-Prot entry P62149). This gene codes for a protein possessing an amino acid sequence identical to human CaM (Uni-Prot entry P62158). The amino acid sequence numbering used in this study (A1-K148) neglects an N-terminal Met because of its cleavage during expression. Before use, the CaM (in 1 mM imidazole, pH 6.5, 0.1 mM KCl and 10 mM CaCl_2_) was exhaustively dialyzed against deionized water to remove all buffer components and Ca^2+^. CaM concentration was determined by using the Lowry method with the Bio-Rad DC protein assay kit with bovine serum albumin as the standard. Aliquots of 60 μM CaM were stored at -80°C.

### Preparation of analyzed small molecules

**Fig 1:**
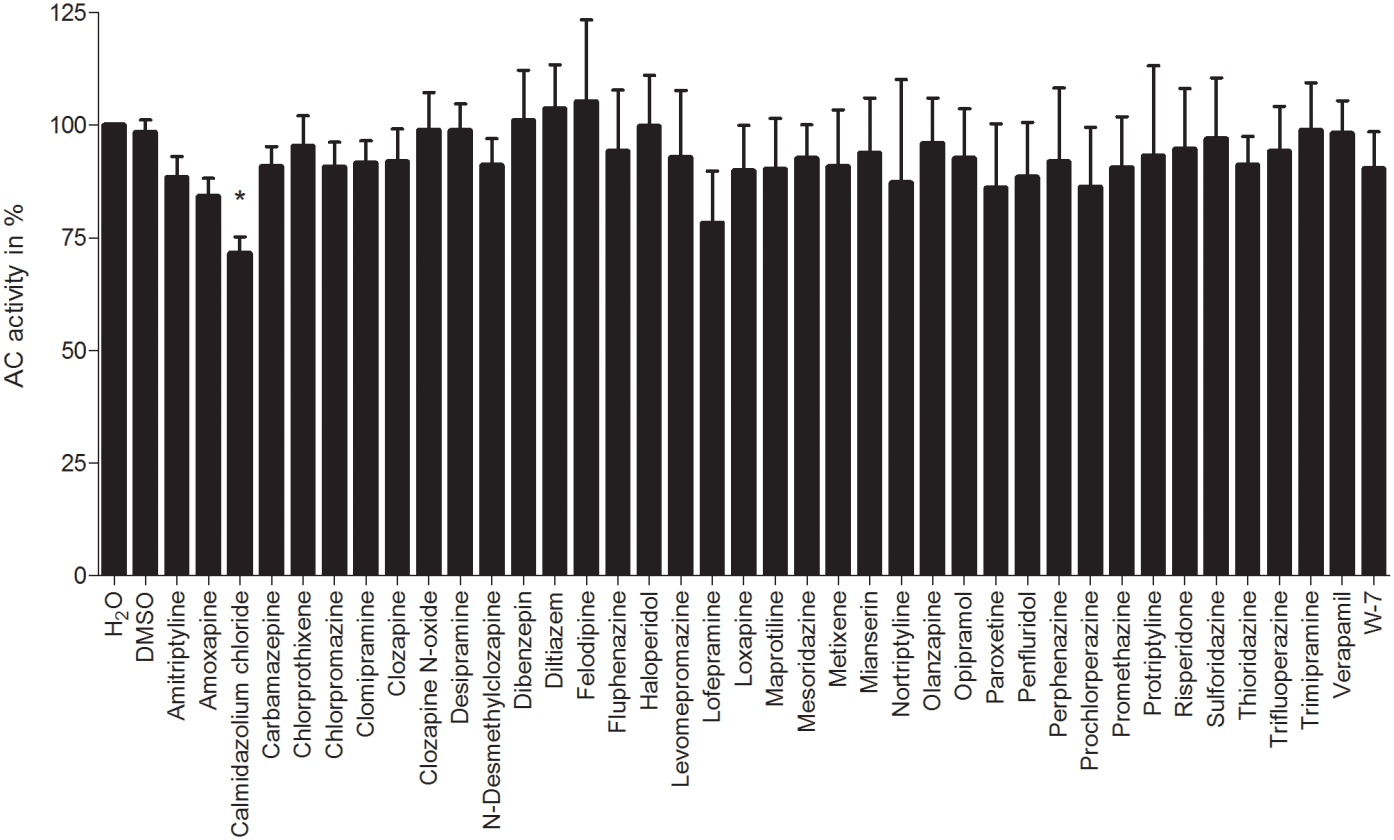
Effects of small molecules on CaM-stimulated activity of AC1. The AC activity assay was performed as described in “Materials and Methods”. A concentration of 1 μM CaM was used for the basal stimulation of AC1. The basal CaM-stimulated AC activity of AC1 was determined with deionized water or 0.1% (v/v) DMSO and was set to 100%, respectively, in accordance to used solvents described in “Materials and Methods”. A concentration of 10 μM of the small molecules was used. The AC activities show the means ± SD of three independent experiments performed in duplicates. A one-way analysis of variances with a Dunnett’s multiple comparison post-test with basal CaM-stimulated AC activity with deionized water or 0.1% (v/v) DMSO, depending on used solvent of each substance, as control was performed to detect significant effects of small molecules on CaM-stimulated AC activity (no*: *p*-value > 0.05; *: 0.01 < *p*-value < 0.05).

**Fig 2:**
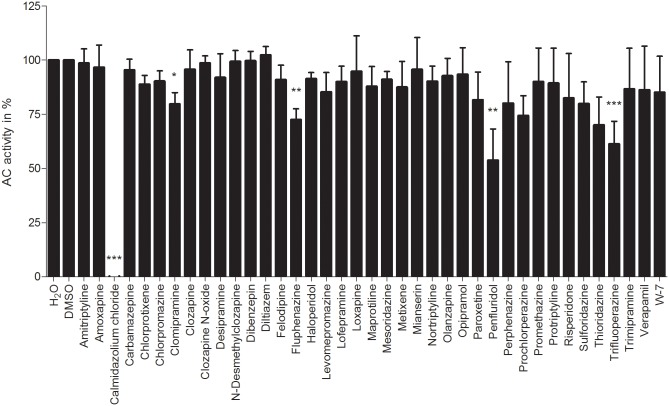
Effects of small molecules on CaM-stimulated activity of EF. The AC activity assay was performed as described in “Materials and Methods”. A concentration of 0.1 μM CaM was used for the basal stimulation of EF. The basal CaM-stimulated AC activity of EF was determined with deionized water or 0.1% (v/v) DMSO and was set to 100%, respectively, in accordance to used solvents described in “Materials and Methods”. A concentration of 10 μM of the small molecules was used. The AC activities show the means ± SD of three independent experiments performed in duplicates. A one-way analysis of variances with a Dunnett’s multiple comparison post-test with basal CaM-stimulated AC activity with deionized water or 0.1% (v/v) DMSO, depending on used solvent of each substance, as control was performed to detect significant effects of small molecules on CaM-stimulated AC activity (no*: *p*-value > 0.05; *: 0.01 < *p*-value < 0.05; **: 0.001 < *p*-value < 0.01; ***: *p*-value < 0.001).

**Fig 3:**
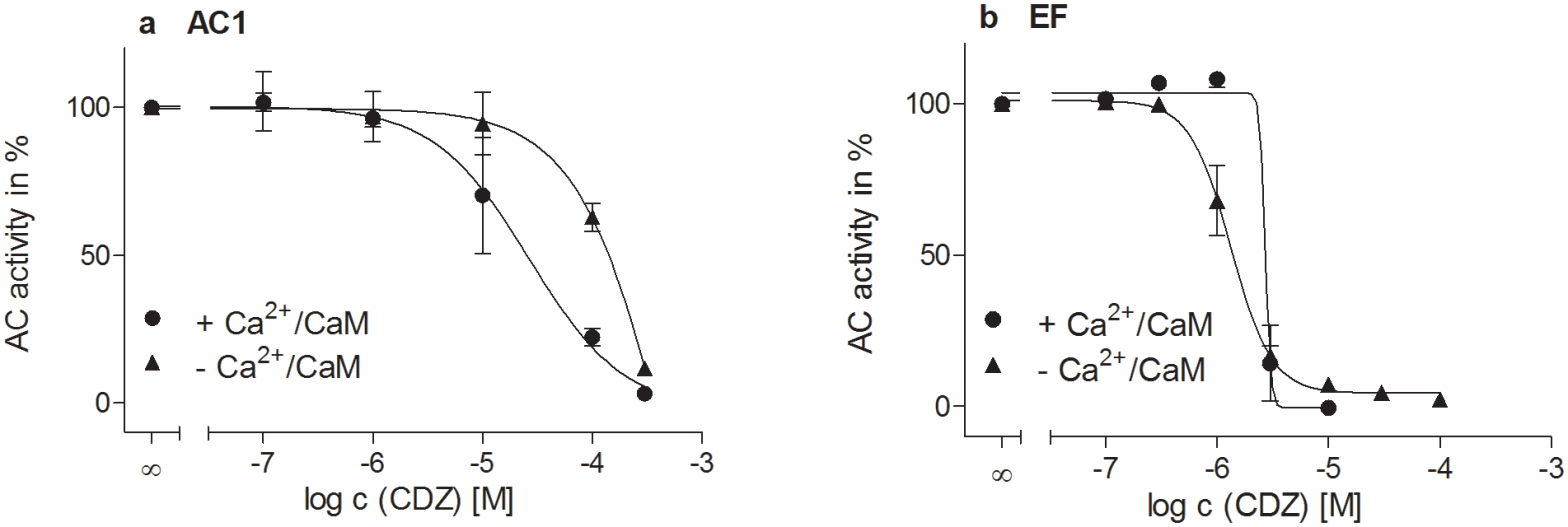
Concentration response curves of increasing concentrations of calmidazolium chloride (CDZ) (0.1–300 μM) with (•) and without (▲) Ca^2+^/CaM for AC1 (a) and EF (b). AC activity was determined as described in “Materials and Methods”. 1 μM CaM was used for experiments with AC1 (a) and 0.1 μM CaM was used for experiments with EF (b). A free Ca^**2+**^-concentration of 10 μM was used in a and b. AC activities are normalized on the basal AC activity of AC1 or EF determined using 1% (v/v) DMSO, respectively. Data show the means ± SD of two (a) or three (b) independent experiments performed in duplicates. All calculations (nonlinear regression (three parameters in case of AC1 and variable slope in case of EF) and normalization) were performed using GraphPad Prism 5.04.

Amitriptyline, fluphenazine, nortriptyline and trifluoperazine were diluted using deionized water (Figs 1 and 2). All other compounds were diluted using (finally) 0.1% (v/v) DMSO because of their lipophilicity (Figs 1 and 2). The compounds were diluted to a final concentration of 10 μM from stock solutions of 10 mM in deionized water or 100% (v/v) DMSO. To determine the concentration response curves shown in Fig 3, CDZ was diluted using (finally) 1% (v/v) DMSO to final concentrations of 0.1 μM to 300 μM. DMSO at used final concentrations did not affect basal AC activity of AC1 or EF (data not shown).

### AC activity assay: Analysis of CaM-stimulated AC activity of AC1

The catalytic activity of AC1 was essentially determined as previously described [21, 54]. Sf9 membranes expressing AC1 were resuspended using syringes at 4°C and diluted with binding buffer (75 mM Tris-HCl, 12.5 mM MgCl_2_ and 1 mM EDTA) to a final protein concentration of 1 μg/μl and a final pH of 7.4. Samples containing 20 μg of protein were pre-incubated for 2 min at 30°C with 10 μl of deionized water or 0.1% (v/v) DMSO (Fig 2) or with 1% (v/v) DMSO (Fig 3) for determining the basal activity. Reactions were initiated by adding 20 μ l of reaction mixture consisting of 10 μM free Ca^2+^, 1 μM CaM, 100 μM cAMP, 40 μM ATP, 100 μM 3-isobutyl-1-methylxanthine, 9 mM phosphocreatine, 0.4 mg/ml creatine kinase, 100 μM EGTA and 0.25 μCi [α-^32^P]ATP to the samples. Additionally, samples contained 5 mM MgCl_2_, 0.4 mM EDTA, 30 mM Tris-HCl, pH 7.4. EGTA was used to keep the concentration of free Ca^2+^ constant. Free Ca^2+^-concentrations, with consideration of buffer components, pH, and temperature, were calculated using WebMax C standard (http://www.stanford.edu/~cpatton/webmaxcE.htm). A concentration of 1 μM CaM-wt was used for the basal stimulation of AC1 because, thereby, a suitable basal AC activity was reached to detect inhibiting or activating effects of small molecules on AC activity. A free Ca^2+^-concentration of 10 μM was used because each CaM molecule binds four Ca^2+^-ions and higher Ca^2+^-concentrations inhibit AC1 activity due to the competition with Mg^2+^ (the physiological ion required for catalytic activity of ACs) at the catalytic site of AC1 [54, 65]. In order to evaluate CaM-independent effects of CDZ on the AC activity of AC1 (Fig 3), a reaction mixture without free Ca^2+^, CaM and EGTA was used. Reactions were conducted for 20 min at 30°C and terminated by adding 20 μl of 2.2 N HCl. Denatured protein was sedimented using a centrifugation for 1 min at 12,000 × g. Separation of the product [^32^P]cAMP from the educt [α-^32^P]ATP was performed by transferring the samples onto columns filled with 1.3 g of aluminium oxide (MP Alumina N Super I). [^32^P]cAMP was eluted from columns with 4 ml of 0.1 M ammonium acetate, pH 7.0. The concentration of [^32^P]cAMP was measured by Čerenkov radiation. The AC activities were calculated after subtracting the blank value, which was determined by transferring only the amount of total added [α-^32^P]ATP on the columns with reference to the amount of total added [α-^32^P]ATP. Blank values were ~ 0.03% and the turnover of substrate was ~ 5% of the amount of total added [α-^32^P]ATP.

### AC activity assay: Analysis of CaM-stimulated AC activity of EF

The AC activity assay to determine the AC activity of EF was essentially performed as described for AC1 with some modifications. Briefly, EF (10 pM, dissolved in 20 μl of 45 mM 2-[4-(2-hydroxyethyl)piperazin-1-yl]ethanesulfonic acid (HEPES), pH 7.4, containing bovine serum albumin (0.1% (m/v)) was pre-incubated for 2 min at 25°C with 10 μl of deionized water or 0.1% (v/v) DMSO for determining the basal EF activity. Reactions were initiated by adding 20 μl of a reaction mixture consisting of 10 μM free Ca^2+^, 0.1 μM CaM, 100 μM cAMP, 40 μM ATP, 100 mM KCl, 5 mM MnCl_2_, 100 μM EGTA and 0.2 μCi [α-^32^P]ATP. Free Ca^2+^-concentrations were chosen and calculated as described for AC1, but with Mn^2+^ instead of Mg^2+^. Reactions were conducted for 10 min at 25°C. A concentration of 0.1 μM CaM-wt was used for the basal stimulation of EF because, thereby, a suitable basal AC activity was reached to detect inhibiting or activating effects of small molecules on AC activity. In order to determine CaM-independent effects of CDZ on the AC activity of EF (Fig 3), reaction conditions were modified to obtain suitable counts per minute for AC activities of EF also in the absence of CaM. For experiments shown in Fig 3 the reaction time was increased to 30 min, the reaction temperature to 30°C, added [α-^32^P]ATP to 0.3 μCi and the EF concentration to 5 nM per sample. The unlabeled amount of ATP was decreased to 20 μM. The reaction mixure was without free Ca^2+^, CaM and EGTA to determine the AC activities of EF in the absence of CaM.

### Statistical analysis

The effects of small molecules on the AC activity of AC1 or EF were compared with one-way analysis of variances with Dunnett’s multiple comparison post-test using GraphPad Prism 5.04 (GraphPad Software Inc., La Jolla, CA, USA). This test was chosen because we were interested in identifying major changes in AC activity rather than small, but statistically significant, effects. AC activity of AC1 stimulated by 1 μM CaM-wt or AC activity of EF stimulated by 0.1 μM with deionized water or 0.1% (v/v) DMSO depending on the solvent of each substance served as controls.

## Results

### Effects of analyzed small molecules on CaM-stimulated catalytic activities of AC1 and EF

The effects of analyzed small molecules on CaM-stimulated AC activity of AC1 are shown in Fig 1 and on CaM-stimulated AC activity of EF in Fig 2. Absolute enzyme activities in the absence and presence of CaM are shown in S1 Fig Stimulation of AC1 and EF was achieved by using 1 μM and 0.1 μM CaM-wt, respectively, to determine an influence of distinct small molecules on CaM-AC1 or CaM-EF interaction. The different CaM-concentrations used for AC1 and EF are caused by their different CaM-affinities. By using the above CaM-concentrations, about half-maximal stimulation was observed for each enzyme. CaM-concentrations of 0.1 μM and 1 μM are physiologically relevant [66, 67].

In contrast to numerous previous studies analyzing the effect of CaM-inhibitors on CaM-target interactions using extremely high concentrations of up to 1000 μ M of CaM-inhibitors (Table 1), a cut-off concentration of 10 μM of each small molecule was chosen for analyses presented in this study. This is because the therapeutic concentration of antipsychotics and antidepressants is generally ≤ 10 μM and results using higher concentrations are not relevant for *in-vivo* conditions [53]. Furthermore, unspecific and CaM-independent effects of CaM-inhibitors on AC1 or EF could be induced in the presence of CaM-inhibitor concentrations above 10 μM. CDZ reduced CaM-stimulated AC1 activity by 28%. All other analyzed compounds failed to influence CaM-stimulated AC1 activity. In contrast to the moderate decrease of AC1 activity by CDZ, this CaM-inhibitor abrogated CaM-activated AC activity of EF. Additionally, clomipramine, fluphenazine, penfluridol and trifluoperazine inhibited the AC activity of EF by about 20%, 30%, 45% and 40%, respectively.

### Inhibition of catalytic activities of AC1 and EF by CDZ in the presence and in the absence of CaM

In order to evaluate if the observed inhibition of the activity of AC1 and EF is CaM-dependent or not, concentration-response curves of the well-known CaM-inhibitor CDZ [48] for AC1 and EF (Fig 3) were determined in the presence as well as in the absence of Ca^2+^ and CaM. The basal AC activities were significantly lower in the absence of CaM, especially in the case of EF (~ 380 pmol cAMP*pmol EF^-1^*sec^-1^ vs. ~ 0.15 pmol cAMP*pmol EF^-1^*sec^-1^) (S1 Fig). The modification of the reaction conditions (described in “Materials and Methods”) resulted in about 200-fold higher counts per minutes for the basal EF activity than the blank values and thus, a valid interpretation of CDZ effects on AC activity of EF in the absence of CaM was possible. The difference between CaM-stimulated and basal AC1 activity was only three-fold (~ 300 pmol cAMP*mg protein^-1^*min^-1^ vs. ~ 100 pmol cAMP*mg protein^-1^*min^-1^) and sufficiently high to study CaM-independent effects of CDZ.

To compare the inhibition effects of CDZ, AC activities were normalized on the basal AC activity of AC1 or EF, respectively, determined using 1% (v/v) DMSO in the presence and in the absence of Ca^2+^/CaM. Both AC1 and EF were inhibited by CDZ in the presence as well as in the absence of Ca^2+^ and CaM. A concentration of 10 μM CDZ decreased the AC activity of AC1 in the presence of Ca^2+^/CaM of about 30% whereas AC1 activity in the absence of Ca^2+^/CaM was not altered at this CDZ concentration. The AC1 activity in the absence of Ca^2+^/CaM was significantly decreased by about 35% at a concentration of 100 μM calmidazoilum chloride. AC1 activity was abrogated in the presence and in the absence of Ca^2+^/CaM at the high concentration of 300 μM CDZ where unspecific effects can occur. Activity of EF was almost inhibited by about 85% at a CDZ concentration of 3 μM both in the presence and in the absence of Ca^2+^/CaM. EF activation was decreased by about 30% in the absence of Ca^2+^/CaM by 1 μM CDZ, whereas in the presence of Ca^2+^/CaM, no inhibition was apparent. The slope of the inhibition curve for EF in the presence of Ca^2+^/CaM was much steeper than in the absence of Ca^2+^/CaM.

## Discussion

Inhibition of AC1 activity via disrupting CaM activation of AC1 could be useful for the treatment of muscle pain, where AC1 supposedly is involved [68]. Otherwise, an improved activation of AC1 may be useful for enhancing processes of memory and learning [6, 9]. Identification of potent small molecules inhibiting CaM-EF interaction is an important goal to develop drugs for the therapy of EF toxinemia. Yet identified EF inhibitors such as “P”-site inhibitors (3'-nucleoside mono-, die and triphosphates), (N-methyl)anthraniloyl-nucleotides ((M)ANT-nucleotides) or adefovir and its active metabolite, targeting the catalytic site of EF, are mostly not selective for EF relative to mammalian ACs and, thus, not clinically useful [69]. In order to investigate both CaM-AC1 and CaM-EF interaction concerning the interference with small molecules, a small library of known CaM-inhibitors and related compounds was analyzed.

The findings with regard to AC1 and EF are essentially in accord with previous findings demonstrating that CaM-AC1 interaction is relatively insensitive to inhibition by small molecules whereas CaM-EF interaction is impaired by particular small molecules [41, 70, 71]. A previous study using rat cerebellar membranes showed an AC activity inhibition of about 70% using 1 μM CaM and 10 μM CDZ [70], but these membranes did not exclusively express AC1, but also other AC isoforms. AC2 and AC9, which are not CaM-stimulated ACs, are also inhibited by CDZ via a CaM-independent allosteric mechanism [51]. This could explain the rather inefficient inhibition of AC1 (28%) observed in this study. The inhibition of AC1 activity in the absence of CaM suggests that AC1 as well as AC2 and AC9 are inhibited by CDZ via an CaM-independent mechanism. CaM-independent inhibition of CaM-target interactions are also known for other CaM-targets such as CyaA, NO-activated sGC, SERCA and MLCK [18,21,49,50,52].

To compare effects of CaM-inhibition by small molecules, it is essential to consider the used CaM-concentration because the higher the CaM-concentration the smaller is the inhibition of CaM stimulated AC1 activity [70], indicating a CaM-dependent inhibition mechanism in addition to the above mentioned allosteric inhibition of AC1. Brostrom et al. observed inhibition of AC activity using rat cerebellar cortex membranes by the phenothiazine antipsychotic chlorpromazine [27]. An involvement of CaM-AC1 interaction in the mechanism of action of these centrally acting antipsychotic drugs is conceivable because of the neuronal expression of AC1. In this study, no AC1 inhibition by chlorpromazine was observed, the discrepancy being explained by the exceedingly high concentration of 100–500 μM chlorpromazine used in the previous study, putatively inducing unspecific and CaM-independent effects [27]. The fact that not even the typical and potent CaM-inhibitors such as trifluoperazine and W-7 inhibit CaM stimulation of AC1 support the hypothesis of a relative refractoriness of CaM-AC1 interaction to inhibition by small molecules.

In contrast to CaM-AC1 interaction, CaM-stimulated EF activity is more sensitive to inhibition by small molecules. CaM stimulation of EF was significantly decreased by the phenothiazine antipsychotics fluphenazine and trifluoperazine, the diphenylbutylpiperidine antipsychotic penfluridol and the imidazolium CDZ. These results are not surprinsing because these small molecules are well-known as potent CaM-inhibitors [26, 48]. Similarly to AC1 inhibition by CDZ, EF inhibition by CDZ proceeds in a CaM-independent manner. In case of EF, the difference in potency of inhibition by CDZ in the presence compared to the absence of CaM is minimal so that an involvement of CaM in the inhibition mechanism is marginal. Moreover, CaM prevents direct EF inhibition by CDZ via inhibitor scavenging because no inhibition was observed using 1 μM CDZ in the presence of CaM, whereas inhibition was evident in the absence of CaM. At higher concentrations of CDZ, inhibition by CDZ prevails over activation by CaM, and EF is also inhibited in the presence of CaM. This suggests that EF is inhibited by CDZ via an allosteric mechanism, without targeting directly CaM-binding to EF. In addition, and more unexpected at first glance, CaM-EF interaction was also inhibited by the tricyclic antidepressant clomipramine. On closer inspection, this finding is not surprising since the dibenzazepine clomipramine possesses the proposed structural features of potent CaM-inhibitors. In detail, the dibenzazepine constitutes the hydrophobic region with two aromatic rings and a positively charged amino group three carbon atoms removed from the ring is also present for mediating electrostatic interactions with the negatively charged CaM [26].

In this study, the positive hit CDZ was chosen as a proof-of-principle to determine whether inhibition of AC activity is mediated CaM-dependently or CaM-independently. To clarify the mode of inhibition in more detail, structure-activity relationships of CDZ derivatives and different concentrations of Ca^2+^ and CaM have to be studied. Furthermore, molecular modelling of the CaM-EF-inhibitor interaction is required. Unfortunately, molecular modelling studies analysing the CaM-AC1 interaction are not possible because the complex has not yet been crystallized.

To identify a lead compound for CaM-EF inhibition, compound libraries have to be screened in high-throughtput assays. This is feasible in case of EF as already demonstrated by Lee et al. [41]. They screened 10,000 compounds using a cell-based assay analyzing cAMP-induced morphological changes in cultered murine adrenocortical cells. Subsequently, an evaluation of promising compounds using a surface plasmon resonance spectroscopy-based assay to detect compounds preventing the association of CaM and EF was performed [41, 61, 72, 73]. Thereby, they identified a compound selectively inhibiting CaM-EF interaction. The selectivity of this compound is caused by binding directly to EF, preventing CaM-binding to EF. CyaA is also inhibited by the compound. This is explained by the similarity of the catalytic domain and the interaction with CaM of CyaA and EF [41, 60, 74–77]. Thus, with regard to the selectivity of CaM-EF inhibitors, it is neceassary to analyze identified potent CaM-EF inhibitors also with respect to diverse other CaM-target interactions in addition to the examined CaM-AC1 interaction. In our study we did not perform intact cell studies with CaM inhibitors. The reason is that CaM inhibitors, specifically CDZ address so many different pharmacological targets that data interpretation becomes impossible [51, 79–83].

Taken together, this study shows how problematic currently available CaM inhibitors are in terms of potency and specificity. Our study also highligths the difficulties to develop potent and selective inhibitors of a specific CaM-target protein interaction. Lastly, great caution must be exerted when attributing inhibitory effects of the prototypical CaM inhibitor CDZ to CaM inhibition. CDZ is highly prone to off-target effects. A review on the critical use of pharmacological inhibitors has been recently published [84].

## Supporting Information

S1 Fig Absolute enzyme activities of AC1 and EF. The AC activity assay was performed as described in “Materials and Methods”. **a**, AC1 activity was determined in the absence or presence of 1 μM CaM. Assays additionally contained CDZ at increasing concentrations. Data shown are the means ± SD of three independent experiments. **b**, EF activity was determined in the presence of 0.1 μM CaM. Assays additionally contained CDZ at increasing concentrations. Data shown are the means ± SD of three independent experiments. **c**, EF activity was determined in the absence of CaM. Assays additionally contained CDZ at increasing concentrations. Data shown are the means ± SD of three independent experiments.
(TIF)
