# Supplementary figures and images for: Effects of 39 Compounds on Calmodulin-Regulated Adenylyl Cyclases AC1 and Bacillus anthracis Edema Factor
Source: PLoS One. 2015 May 6;10(5):e0124017. doi: 10.1371/journal.pone.0124017 (PMC4422518; doi:10.1371/journal.pone.0124017)

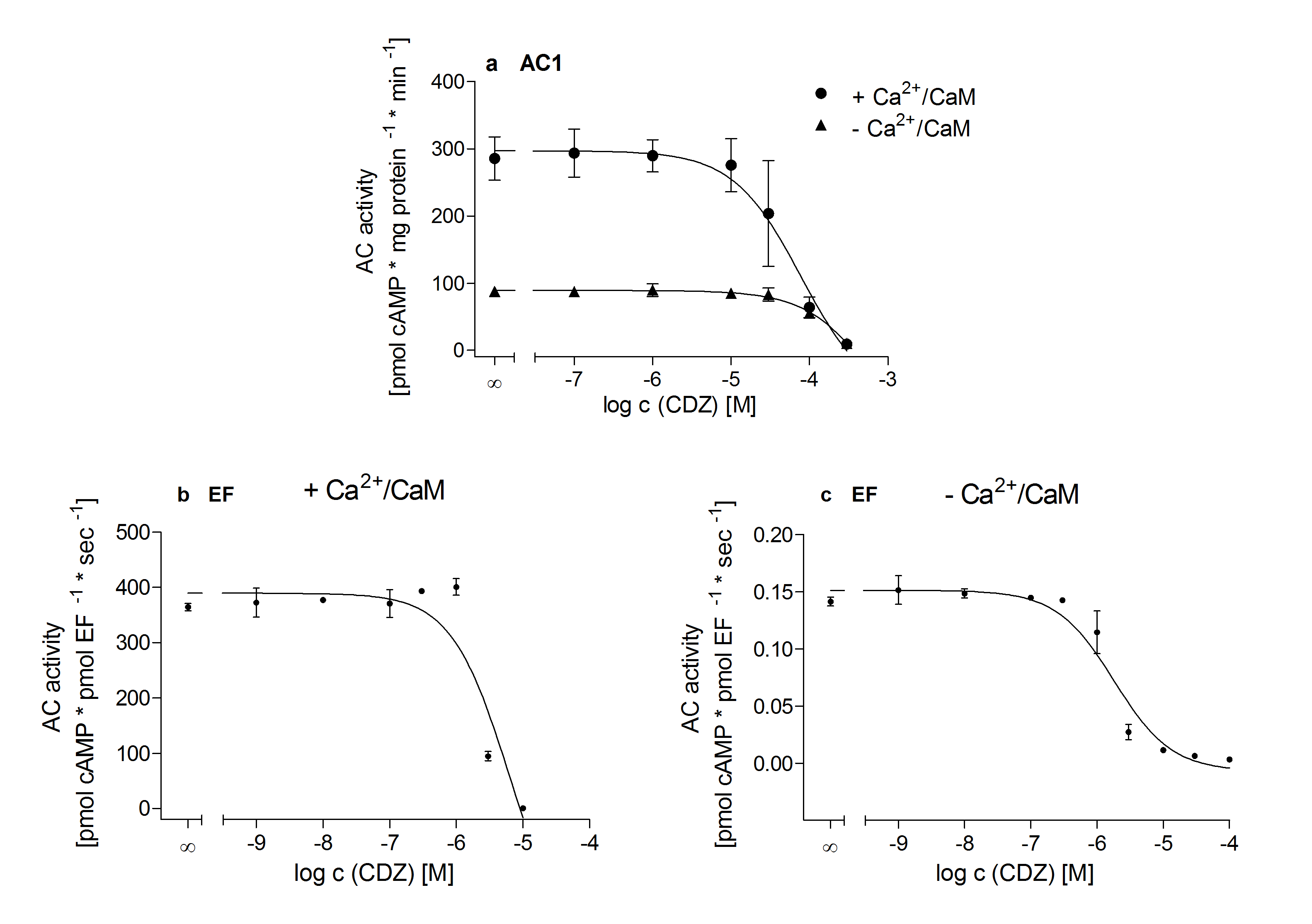

Supplement: S1 Fig — The AC activity assay was performed as described in “Materials and Methods”. a, AC1 activity was determined in the absence or presence of 1 μM CaM. Assays additionally contained CDZ at increasing concentrations. Data shown are the means ± SD of three independent experiments. b, EF activity was determined in the presence of 0.1 μM CaM. Assays additionally contained CDZ at increasing concentrations. Data shown are the means ± SD of three independent experiments. c, EF activity was determined in the absence of CaM. Assays additionally contained CDZ at increasing concentrations. Data shown are the means ± SD of three independent experiments. (TIF) [file pone.0124017.s001.tif]
